# Ethnomycological knowledge in three communities in Amealco, Quéretaro, México

**DOI:** 10.1186/s13002-017-0202-7

**Published:** 2018-01-26

**Authors:** Daniel Robles-García, Humberto Suzán-Azpiri, Adriana Montoya-Esquivel, Jesús García-Jiménez, Edgardo Ulises Esquivel-Naranjo, Elhadi Yahia, Fidel Landeros-Jaime

**Affiliations:** 1 Laboratorio de Ecología y Sistemática de Microorganismos, Facultad de Ciencias Naturales, Universidad Autónoma de Querétaro, Santiago de Querétaro, Querétaro, Mexico; 2 Laboratorio de Ecología, Facultad de Ciencias Naturales, Universidad Autónoma de Querétaro, Santiago de Querétaro, Querétaro, Mexico; 3 Centro de Investigación de Ciencias Biológicas, Universidad Autónoma de Tlaxcala, Tlaxcala, Mexico; 4 Instituto Tecnológico de Ciudad Victoria, Ciudad Victoria, Tamaulipas, Mexico; 5 Laboratorio de Microbiología Molecular (LAMIMO), Facultad de Ciencias Naturales, Universidad Autónoma de Querétaro, Santiago de Querétaro, Querétaro, Mexico; 6 Laboratorio de Fitoquímicos y Nutrición Humana, Facultad de Ciencias Naturales, Universidad Autónoma de Querétaro, Santiago de Querétaro, Querétaro, Mexico

**Keywords:** Ethnomycology, México, Otomí, Cultural Significance Index

## Abstract

**Background:**

Fungi have multiple uses in temperate areas of México, but an important decrease in the traditional knowledge of uses and customs of mushrooms becomes a fundamental issue for fungi conservation. However, only few studies quantify the traditional ethnomycological knowledge in México, and this study is the first quantitative report for Querétaro, a central state with both Otomí and Mestizo communities and a high fungi diversity.

**Methods:**

The present study was conducted registering traditional knowledge on the use and consumption of mushrooms in three *Hñähñu* (Otomí) communities (Tesquedó, Xajay, and Tenasdá) in Amealco de Bonfil, Querétaro, México, between August 2013 and November 2014. We conducted a stratified sampling, where uses common *Hñähñu* and Spanish names, and eight quantitative variables that conform the “Edible Mushrooms Cultural Significant Index” (EMCI) were recorded from 100 informants. For the classification and ordination analysis of species and uses, we used multivariate techniques such as cluster, multidimensional scaling, and principal components (PC).

**Results:**

Thirty-three mushrooms species were registered, most of them used for consumption by households, few aimed for commercial purposes, one species is medicinal, another has veterinary, and other ludic uses (as a toy). The three species with the highest EMCSI were *Amanita basii*, *Fistulinella wolfeana,* and *Lactarius indigo*. Edibility was the main use detected in the survey, and people harvested mushrooms provided by the forest mainly during the rainy season. We observed that mushroom searching and collection are activities that strengthen the family ties and are crucial for the transfer of this knowledge through generations. Cluster analysis separates groups according to different values in EMCSI variables, and principal components ordinate the species by frequencies (PC1) and traditions (PC2).

**Conclusions:**

The current state of knowledge in the studied communities is strong, especially among women, but with a tendency to disappear due to migration and lack of interest among new generations. Future quantitative studies are important to analyze tendencies of the traditional ethnomycological knowledge transferred to new generations.

## Background

The first ethnomycological studies in México were conducted with emphasis on mushrooms used in rituals, but indigenous knowledge goes beyond this use, such as species used for food, medicine, and recreation [1–4].

With the arrival of the Spaniards, many of these uses and customs were banned, because these traditions were considered as inappropriate [5]. Responding to this cultural loss, ethnomycological studies have taken great importance, since they have helped to rescue cultural knowledge and practices, not only on mushrooms but on different natural resources that have survived in many indigenous groups [6, 7]. Fungi play an important role as a livelihood mechanism during the rainy season [5, 8–10], and women are the key element in the transmission of this knowledge [11]. In addition, mushrooms are important in the long-term conservation process as a high-value non-timber resource [12].

Data from different sources shows that indigenous people collect fungi mainly in the rainy seasons for direct consumption by households, while mestizo people in México use them to obtain monetary benefits by selling them in municipal markets [12] or house by house, which is a local activity called “rancheo,” which generates an alternative way to obtain economic incomes [9, 13]. It is important to point out that consumption and selling are not exclusive activities of communities in temperate zones but in tropical areas too [10, 14].

Traditional knowledge on the recognition of edible wild mushrooms is very important for the implementation of adequate management strategies and for the transfer of the mycological knowledge to the new generations. Therefore, inadequate practice or ignorance about such species can lead to the use of toxic fungi and then health consequences such as irreversible damage of the vital organs, such as liver and kidneys [15].

The traditional knowledge on forest resources can be measured based on how many times it is mentioned by people [11, 12, 16–18] or by the use value according to how many ways it can be used [19]; nevertheless, there are other quantitative methods, such as the “Cultural Significance Index,” first developed for plants [20, 21] and then modified for mushrooms [22]. This index is used to analyze several variables in a specific region to detect those plants or mushrooms that people consider the most valuable [21–23] and can be adjusted or adapted to each specific case [24–26].

Mycological studies in México with *Hñähñu* communities have been carried out mainly in two federal estates “Estado de México” and “Hidalgo” [9, 10, 24–29]. However, there are *Hñähñu* settlements in other states such as Guanajuato, Jalisco, Michoacán, Puebla, Querétaro, Tlaxcala, and Veracruz [30]. The state of Querétaro is located in the center of México, and the municipality of Amealco has several *Hñähñu* population settlements, with important interactions with the *Hñähñu* from the neighbor state “Estado de México” where Núñez-López [31] reported that the region is rich in fungi diversity, and that this ethnic group considered them as plants. However, the scarcity of ethnomycological data for the region and for this ethnic group is important [22].

The aim of this study is to answer some fundamental questions about the real knowledge of fungi by the *Hñähñu*, such as the recognition of mushrooms as a biological group, the recognition of different species and its edibility, medicinal or dangerous properties, and finally how and who are the responsible for the transference of mycological knowledge to the next generations, using interviews and participant observation. A technique that helps us to register and quantify different assets of the traditional knowledge is the Cultural Significant Index because it provides an objective numerical scale, eliminating the subjectivity of giving arbitrary values to each variable. This scale was initially proposed by Pieroni et al. [21] as the “Cultural Food Significant Index” for edible plants in northwestern Tuscany, Italy, and modified to edible mushrooms by Garibay-Orijel et al. [22] that complemented with multivariate analysis could help us to answer those questions.

The two main objectives of this study are (a) to conduct the first ethnomycological study in three *Hñähñu* communities in Querétaro, México, and (b) to make the first quantitative analysis of ethnomycological knowledge in Querétaro, using the “Cultural Food Significant Index.”

## Methods

### Location

Amealco is one of the 18 municipalities in the state of Querétaro, located southeast of the capital, between the coordinates 20° 11′ 17″ N and 100° 8′ 38″ W, with an altitude between 2500 and 3150 m above sea level [32], and with an annual rainfall of 500–800 mm [33]. Tesquedó, Xajay, and Tenasdá communities are located at the base of Peña de Ñado, a rock formation that belongs to the province of the Trans-Mexican Volcanic Belt, and consists mainly of oak forests, sometimes combined with *Arbutus* (“madroño”), *Arctostaphylos* (“pingüica”), and pine [34, 35].

### Population

Querétaro has the third biggest community *Hñähñu* speaking Otomí in México: 18,933 inhabitants, of whom 11,740 (62%) live in Amealco [36]. The three selected communities were Tesquedó, Xajay, and Tenasdá, which have a population of 190, 418, and less than 600 inhabitants, respectively [30], and due to the proximity to the forest.

### Ethnographic work

To determine the used species of mushrooms, we conducted a stratified sampling [37]. The people interviewed were 70 women and 30 men, 18 of them from Tesquedó, 32 from Xajay, and 50 from Tenasdá (Table 1). Additionally, to obtain more information, we used two techniques: “participant observation” and “snowball technique.” The first aim is creating a trust atmosphere and the second is to find the main informants [38]. Informal interviews were conducted during the dry season. In the interviews, we showed them photographs of mushrooms, so they could recognize those they use for consumption or medicinal purposes. Questions considered all sub-indexes of the Cultural Significant Index (CSI) proposed by Garibay-Orijel et al. [22] for mushrooms. During the rainy season, mushrooms were collected with key informants in each community to find the species more utilized. In addition, local markets were visited to record which mushrooms are sold and their value. Hospitals were also visited to investigate reported cases of mushroom poisoning. Finally, the purpose of the study in the three communities was descriptive and not comparative since the small number of informants and the relatively similar environments for the three human populations.

**Table 1:** Population interviewed by gender and age in Amealco, Querétaro, Mexico

Gender	Community					
	Tesquedó		Xajay		Tenasdá	
	Interviewed	Age range	Interviewed	Age range	Interviewed	Age range
Male	6	28–63	13	26–75	11	32–74
Female	12	21–62	20	18–73	38	19–72

### Edible Mushrooms Cultural Significant Index

The Garibay-Orijel et al. [22] methodology has been followed to determine the Edible Mushrooms Cultural Significant Index (EMCSI), using the following equation:

$$ \mathrm{EMCSI}=\left(\mathrm{QI}\times 10\right)+\mathrm{PAI}+\mathrm{FUI}+\mathrm{TSAI}+\mathrm{MFFI}+\mathrm{KTI}+\mathrm{HI}+\mathrm{EI} $$

Where:

- QI, the quotation or citation index is a sub-index that relates the number of times a fungus is mentioned, divided between the number of informants, and by tenfold (to keep the scale of the other sub-indexes).
- PAI, the perceived abundance index, is the abundance of each species of mushroom that people can perceive during the rainy season, how many of these species they can find, and how many they can collect.
- FUI, Frequency of Use Index, is how often people consume each species of mushrooms during the rainy season.
- TSAI, taste score appreciation index, using the scale to evaluate the taste of mushroom species consumed by people.
- MFFI, multifunctional food index, to evaluate how people cook mushrooms and whether they eat them alone or combined.
- KTI, knowledge transmission index, to determine how knowledge is transmitted or how people learn how to use the mushrooms.
- HI, Health Index, indicates how safe people feel to consume these mushrooms, and how beneficial is for their health.
- EI, Economic Index, indicates if people sell mushrooms

### Multivariate data analysis: cluster of species and sub-indexes, multidimensional scaling, and principal components analysis

For the classification analysis of species (rows) and sub-indexes (columns) in a multivariate matrix, a cluster analysis was conducted with standardized Euclidean distances (SED) and the Ward clustering method using JMP 8 for Mac (SAS Institute, Cary, NC). For the ordination methods (MDS and PCA), a matrix containing the 33 species mentioned in the interviews and the averages of each sub-index were constructed and analyzed with PC-ORD 6.08 [39] methods. Multidimensional scaling (MDS) was conducted to find grouped species based on the similarity of their sub-indexes values. A principal components analysis (PCA) was also carried out considering the eigenvectors with values higher than 0.46 (positive or negative) between the eight sub-indexes and all the species, in order to find out which are the main sub-indexes in this survey.

### Collection and determination of species

Specimens were collected from April 2013 to November 2014. The sampling was conducted in forests dominated by *Quercus* spp. and *Pinus* spp. according to the methods proposed by Guzmán [40]. Photographs of fresh material were taken with a semi-professional Nikon D3000 camera. Species identification according to specialized literature [41–47] and specialty items were used when necessary. The specimens were dried and deposited in the Laboratory of Systematic Ecology and Microorganisms (Laboratorio de Sistemática y Ecología de Microorganismos), Autonomous University of Querétaro (Universidad Autónoma de Querétaro) (Appendix).

## Results and discussion

People from the three communities still conserve the tradition of collecting mushrooms, mostly for self-consumption and some for sale, depending on the abundance. We recorded 155 Spanish common names and 21 Otomí common names (Table 2), and “hyethe” was the main word used to refer to mushrooms, that means “in rainy season” or jo (that means “sponge”) as Núñez-López [31] reported for some species. A total of 33 mushrooms species had ethnomycological value (Table 3), mainly Boletes [22] and Agarics [17].

**Table 2:** Correspondence between scientific and folkloric name

Division	Family	Species	Otomí name	Spanish name(s)
Ascomycota	Helvellaceae	*Helvella crispa*	*Hyethe de mejcua*	*Hongo Mijcua* (*Conejo*)
	Hypocreaceae	*Hypomyces lactifluorum*	*Xiñu dega thu’tsi*	*Trompas rojas, hongo trucha, trompa de puerco, truchas coloradas, trompitas de puerco, truchas de puerco*
Basidiomycota	Agaricaceae	*Agaricus campestris*	w/r	*Hongo de llano, champiñón de llano, blanco, blanquito, champiñoncito*
		*Calvatia cyathiformis*	*Hyethe bola*	*Hongo bola, de lagartija, de ternera, de llano, sirena, de ternera, quesadilla, canelita, Bolita de llano, llanero, sirenita, patarata, serena, llanerita, canelitas, bolita, bolita de campo, bola*
		*Lycoperdon marginatum*	w/r	*Hongo de camaleón, de sangra, sirena*
		*Lycoperdon perlatum*	*Hyethe tsíja*	*Hongo bolita, de bola, sangano, quesadilla, hongo sirena, serenas, de lagartija*
	Amanitaceae	*Amanita basii*	*Gshmu*	*Cashimón, hongo Santiago, cashimoses, cashimones, yema, amarillo*
		*Amanita novinupta*	w/r	*Hongo Santiago, santiaguero*
	Boletaceae	*Boletus auripes*	*Hyethe de ndega*	*Hongo de buey, de manteca, amarillo*
		*Boletus* aff. *speciosus*	*Hyethe kjoboy*	*Hongo de res, de buey, vaca, hongo joboy, joboy*
		*Boletus* sp.	*Hyethe kjoboy*	*Hongo de buey, de vaca, de res, joboy*
		*Boletus variipes*	*Hyethe nt’axi*	*Hongo Blanco, de buey, de buey blanco*
		*Exudoporus frosti*	*Hyethe kjoboy ntheni*	*Hongo de buey, de buey rojo, joboy rojo, de madroño*
		*Fistulinella wolfeana*	*Ushki hyethe*	*Hongo salado, dulce, pansza*
		*Harrya chromapes*	w/r	*Hongo de vivora, blanco, de pingüica, de madroño, madroñito*
		*Leccinum* aff. *aurantiacum*	*Hyethe dega penxi*	*Hongo de pingüica, de pendicua, de madroño*
		*Leccinum rugosiceps*	*K’ast’i hyethe*	*Hongo amarillo, escobilla, de buey, de manteca*
		*Leccinum* sp.	w/r	*Negrito, blanco, negro, sacatón, de trigos, de trigo*
		*Retiboletus* aff. *griseus*	*Hyethe ngut’ei (dega ñoñxu)*	*Negrito, de pasto, sacatón, sacatoncito*
		*Suillus granulatus*	*Hyethe dega tgu’di*	*Hongo de pino, de madroño, de ocote, ocote, sacatón, baboso, agrio, panza de ocote, pancitas, pancita de ocote, de coshal (hojarasca de pino)*
		*Xerocomus illudens*	*Ixka hyethe*	*Hongo de azufre, agrio, amarillo, de madroño*
	Cantharellaceae	*Cantharellus cibarius group*	*Hyethe mijkwa*	*Pericón, Santa Mária*
	Entolomataceae	*Nolanea* sp.		*Hongo de rayo*
	Gomphaceae	*Ramaria* spp.	*Hyethe ts’ints* ***u***	*Patitas de pájaro, pata de pájaro, hongo pájaro, hongo pata de pájaro, pata amarilla, pata de pájaro blanca*
	Physalacriaceae	*Armillaria mellea*	*Hyethe dega nd* ***u*** *nza*	*Hongo de tronco, de hoja, amarillo, clavo, de rama, de palo, de hojarasca*
	Russulaceae	*Lactarius indigo*	*Guilloi (ñäi)*	*Zorrillo, zorrillo azul, hongo pantalón, trompas azules, hongo azul, hongo de zorrillo*
		*Russula* aff. *brevipes*	w/r	*Hongo de borrega, trompas blancas*
		*Russula* aff. *cyanoxantha*	w/r	*Hongo de borrega, hongo de madroño*
		*Russula* aff. *lepida*		*Hongo de madroño, hongo de borrego*
	Tricholomataceae	*Clitocybe gibba*	*Hyethe de gashisa (hanxiza)*	*Hongo de hoja. Clavito, tejamanil, delgado, truchita, corralito, de fumador, de copa, de hojarasca, montonero*
	Marasmiaceae	*Gymnopus driophilus*	w/r	*Hongo de hoja, sombrerito, hongo niña, montonera, corralitos, hongo de rayo*
	Ustilaginaceae	*Ustilago maydis*	w/r	*Hongo de maíz, de elote, huitlacoche*

*w/r* without record

**Table 3:** Cultural significance values for edible wild fungi in Tesquedó, Xajay, and Tenasdá communities in Amealco, Querétaro, Mexico

No.	Species	QI	PAI	FUI	TSAI	MFFI	KTI	HI	EI	EMCSI
1	*Lactarius indigo*	8.70	9.91	9.34	9.07	9.32	10.00	6.67	0.23	63.24
2	*Amanita basii*	9.30	8.06	9.06	9.89	8.49	10.00	6.67	0.47	61.95
3	*Fistulinella wolfeana*	8.30	8.92	9.31	8.39	9.12	10.00	6.67	0.52	61.23
4	*Rammaria* spp.	8.00	8.59	8.53	9.08	8.71	9.97	6.67	0.29	59.85
5	*Boletus variipes*	7.50	8.63	8.90	9.31	7.06	10.00	6.67	0.31	58.38
6	*Boletus* aff. *speciosus*	6.70	7.69	8.36	9.11	8.18	9.78	6.67	0.15	56.52
7	*Leccinum* aff. *aurantiacum*	8.00	6.78	7.72	8.71	7.94	9.94	6.67	0.37	56.13
8	*Hypomyces lactifluorum*	8.20	6.65	6.46	9.03	8.60	10.00	6.67	0.33	55.93
9	*Calvatia cyathiformis*	6.30	6.90	7.78	9.10	8.15	9.84	6.67	0.26	54.90
10	*Armillaria mellea*	6.30	8.85	7.10	8.94	6.85	9.88	6.67	0.16	54.75
11	*Ustilago maydis*	4.40	7.67	8.41	9.47	8.32	9.77	6.67	0.00	54.56
12	*Clitocybe gibba*	4.60	7.88	6.79	9.64	7.89	10.00	6.67	0.14	53.62
13	*Agaricus campestris*	3.50	7.64	7.64	9.52	7.89	9.71	6.67	0.95	53.34
14	*Russula* aff. *cyanoxantha*	1.00	10.00	7.75	8.67	8.85	10.00	6.67	0.00	52.94
15	*Retiboletus* aff. *griseus*	5.40	8.15	7.78	8.13	6.52	9.95	6.67	0.00	52.60
16	*Boletus auripes*	5.60	5.94	6.74	9.17	7.51	10.00	6.67	0.12	51.74
17	*Leccinum* sp.	5.10	7.79	6.91	7.57	6.35	9.95	6.67	0.00	50.35
18	*Suillus granulatus*	4.80	9.06	6.46	7.08	6.66	9.79	6.46	0.00	50.32
19	*Russula* aff. *brevipes*	1.00	9.00	8.25	7.00	8.40	9.50	6.67	0.00	49.82
20	*Boletus* sp.	2.10	7.50	7.86	8.26	8.17	9.52	6.67	0.00	49.76
21	*Harrya crhomapes*	5.20	6.59	7.02	7.93	5.88	10.00	6.67	0.00	49.29
22	*Lycoperdon marginatum*	1.60	7.97	5.00	9.17	8.81	10.00	6.67	0.00	49.22
23	*Lycoperdon perlatum*	2.00	6.13	6.88	9.17	8.33	10.00	6.67	0.00	49.16
24	*Exudoporus frostii*	1.20	8.75	8.33	8.61	6.13	9.17	6.67	0.00	48.86
25	*Cantharellus cibarius*	3.60	6.25	6.18	8.98	7.15	9.72	6.67	0.19	48.56
26	*Nolanea* sp.	1.00	6.50	6.25	9.33	8.70	10.00	6.67	0.00	48.45
27	*Russula* aff. *lepida*	2.90	8.62	6.64	6.32	7.45	10.00	6.44	0.00	48.37
28	*Lyophyllum* sp.	0.30	6.67	6.67	10.00	8.00	10.00	6.67	0.00	48.30
29	*Leccinum rugosiceps*	4.00	7.00	7.88	8.23	4.08	10.00	6.67	0.00	47.85
30	*Xerocomus illudens*	5.30	6.42	7.08	6.67	5.71	10.00	6.67	0.00	47.84
31	*Gymnopus dryophilus*	1.50	5.33	5.50	9.78	8.70	10.00	6.67	0.00	47.48
32	*Amanita novinupta*	0.60	5.00	6.67	8.34	3.33	10.00	6.67	0.00	40.61
33	*Helvella crispa*	0.10	2.50	2.50	10.00	7.50	10.00	6.67	0.00	39.27

*QI* Mention Index, *PAI* perceived abundance index, *FUI* Frequency Of Use Index, *TSAI* taste score appreciation index, *MFFI* multifunctional food index, *KT*I knowledge transmission index, *HI* Health Index, *EI* Economic Index, *EMCSI* Edible Mushrooms Cultural Significance Index

### EMCSI sub-indexes

**Fig. 1:**
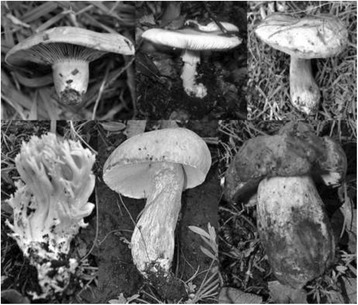
From left to right and top to bottom, the six most valuable species in Tesquedo, Xajay and Tenasdá communities, Amealco, Querétaro, Mexico: *Lactarius indigo*, *Amanita basii*, *Fistulinella wolfeana*, *Ramaria* sp., *Boletus variipes*, and *Boletus* aff. *speciosus*

Citation Index (QI). *Amanita basii* Guzmán & Ram.-Guill., *Lactarius indigo* (Schwein.) Fr. and *Fistulinella wolfeana* Singer & J. García, which was the first record of edibility [47], were the most mentioned species during the surveys; these results are comparable to those of Alonso-Aguilar et al. [23] where *A. basii* is the most important mushroom in San Mateo Huexoyucan, Tlaxcala, México, and Montoya et al. [16] found it only by using a free list. We can argue that most of the time, the first mushroom cited is the most important. In contrast, other genus such as *Ramaria* Fr. ex Bonord is considered the most important to people of La Laguna de Fúquene, Andes Nororientales [26]. Here, *Helvella crispa* (Scop.) Fr. was mentioned to be consumed by only one person. *Lyophyllum* P. Karst had few mentions, which might be because only few people know where to find it [29] (Fig. 1).

perceived abundance index (PAI). *Lactarius indigo*, *Russula* aff. *brevipes* Peck, *R*. aff. *cyanoxantha* (Schaeff.) Fr., and *Fistulinella wolfeana* had the highest values, while *Amanita novinupta* Tulloss & J.E. Lindgren, *Boletus auripes* Peck, *Gymnopus dryophilus* (Bull.) Murrill, *Helvella crispa*, and *Suillus granulatus* (L.) Roussel had the lowest values. It is important to point out that *Russula* and *Agaricus campestris* L. were the most abundant according to with Peña-Cañón & Enao-Mejía [26], while *A. campestris* is the most abundant for Alonso-Aguilar et al. [23]; this result could be linked to the vegetation or the season where the study was carried out. Even when *Russula* spp. or *S. granulatus* is common in the forest, people preferred to collect other mushrooms because they do not consider these palatable. Sometimes, when *Ramaria* spp. is not common, people prefer to walk long distances to find it. In this case, as mentioned by Bautista [29], some species such as *A. basii* are more difficult to find due to the condition of forests. Some informants mentioned that the number of mushrooms they could find is decreasing, arguing that the abundance and uses were higher in the past.

Frequency of Use Index (FUI). *Amanita basii*, *Boletus* spp., *Fistulinella wolfeana*, *Lactarius indigo*, *Ramaria* spp., and *Ustilago maydis* (DC.) Corda are consumed four times, or more, during the rainy season, and sometimes even four times a week, depending on their abundance. Peña-Cañón and Enao-Mejía [26] and Alonso-Aguilar et al. [23] mentioned that the most commonly used fungi were *Russula* sp. and *A. basii*, and Garibay-Orijel et al. [22] said that *Cantharellus* Juss and *Pleurotus* (Fr.) P. Kumm. are the most abundant mushrooms in Ixtlán de Juárez, Oaxaca. *Hypomyces lactifluorum* (Schwein.) Tul. & Tul. has a minor index, probably because people have to scrape the ground to find it. Less-used mushrooms were *Helvella crispa*, *Gymnopus dryophilus*, and *Lycoperdon marginatum* Vittad.; the first is due to its low abundance, the second because only some people consider it as non-edible, and the third is due to the need of finding a considerable number of individual mushrooms to prepare a hearty meal. It is important to note that the frequency of use of determined species is determined by the access and the amount available.

Taste score appreciation index (TSAI). In general, all mushrooms were well accepted by people who consume them; *Amanita basii* was the most palatable species according to many informants as we could see with Alonso-Aguilar et al. [23]. In contrast, species of the genus *Ramaria* were the most appreciated according to Peña-Cañón and Enao-Mejía [26], and *Gomphus clavatus* (Pers.) Gray had the highest value of the overall species reported by Garibay-Orijel et al. [22]. Only few species were not appreciated, such as *Harrya chromapes* (Frost) Halling, *Russula* aff. *lepida* Fr., *Suillus granulatus*, and *Xerocomus illudens* (Peck) Singer, mainly because some *Russula* species have a spicy flavor, and some have a sour taste, such as the case of these boletes.

Multifunctional food index (MFFI). The main form of mushroom consumption was by roasting, only by cooking on a griddle (comal), with a little salt. Many people cook them with green sauce or “pasilla” chili sauce. The only mushrooms consumed alone were *Amanita basii*, *Ustilago maydis*, *Boletus auripes* Peck, and *Hypomyces lactifluorum* for being very palatable. *Fistulinella wolfeana* and *Lactarius indigo* can only be eaten roasted and have fewer larvae inside. Some people like to mix mushrooms, and the most frequent combination include *Cantharellus cibarius* Fr., *Boletus variipes* Peck, and *Lactarius indigo*, referring this as a very delicious combination. *Calvatia cyathiformis* (Bosc) Morgan and *Agaricus campestris* could also be consumed mixed together because they have a meat-like taste. *Lycoperdon perlatum* Pers. and *L. marginatum* were also consumed alone, as *U. maydis* in “quesadillas,” but the latter could be eaten mixed with squash blossoms. *Ramaria* spp. could be prepared alone too, and in many cases with eggs. Some mushrooms can be preserved for long periods, such as *L. indigo* and *F. wolfeana*, which are dried on rocks in the sun, or hanged on a chain, as reported by Estrada-Torres and Aroche [7]. *Ramaria* spp. is shredded into fine strands, and *Hypomyces lacrifluorum* is cut into thin slices and left in the sun, sometimes preserved frozen for up to 3 months. Our results on the MFFI are consistent with those of Garibay-Orijel et al. [22], Alonso-Aguilar et al. [23], and Peña-Cañón and Enao-Mejía [26] where we can see a close relationship with this sub-index and the TSAI sub-index, but in every case, the way of cooking varies.

Knowledge transmission index (KTI). Perhaps, this is the main index of the survey because it allowed to detect how traditional knowledge has been transferred from one generation to another. Méndez et al. [48] and Garibay-Orijel et al. [49] reported that women play a key role in transmitting and preserving knowledge because men were working out of town most of the time and only spend a few days at home. However, knowledge is diverse. We found that some people knew more than 30 different mushrooms, while some others only knew 3 species. In many cases, people refer to some species that were consumed by their parents or grandparents, but they do not eat them actually, which eventually represents the loss of this knowledge. Some younger people have not shown interest in learning the traditional uses of fungi. The ethnic group with the smaller ethnomycological knowledge was the mestizos, while Otomí people usually detected and use more fungi species in the area. Some species, such as *Exudoporus frostii* (J.L. Rusell) Vizzini, Simonini & Gelardi or *Boletus* cf. *speciosus* Frost, were considered poisonous by some informants, because they have blue spots (like other boletes that change color); however, a woman (and her family) who learned that *E. frostii* is edible, has shared that knowledge with their neighbors, situation previously observed by Garibay-Orijel et al. [22] and Peña-Cañón and Enao-Mejíar [26] too. For most species, knowledge can be traced for 50 years through two or three generations. It was also noted that discrimination plays an important role since some people argued that mushrooms are the “food of the poor.” As Burrola-Aguilar et al. [27] mentioned, in the case of fungi, many of the knowledge were lost because people migrated to national or international (mainly US) urban areas for better working conditions and the pursuit of economic improvements, resulting in trans-cultural and adoption of Western model over their culture and traditions [29].

Mushroom collection is commonly a family activity, in which people share and strengthen their family ties, and it is where children (from 5 to 10 years old) learn how to recognize edible and inedible mushrooms, and where mushrooms grow (main collection points). People have their techniques to recognize edible specimens from inedible ones, but it has been mentioned that some inedible species were similar to edible mushrooms. People commonly collect only red *Russula* species, which do not have a spicy taste, or *Ramaria* that have a mild flavor. Regarding the names of the mushrooms, most of them are similar to those reported elsewhere and are consistent with recent studies in Otomí areas [25–29].

Health Index (HI). People indicate that when they eat mushrooms, they cannot drink milk or alcohol, eat avocado, or consume medicine because they have side effects. Only *Russula* aff. *lepida* and *Suillus granulatus* were consumed with caution. The “skin hat” is commonly removed because it is considered as the main factor causing diarrhea or stomach pain. *Xecomus illudens* and *Harrya chromapes* are reported to have a sour taste but without consequences. Besides, only some people mentioned feeling healthier by eating mushrooms in general [22], and not only with the consumption of some species, they generally refer to assimilating minerals from the soil through eating mushrooms. It is important to not confuse these with the medicinal proprieties of used fungi, and finally, they think that mushrooms are better than meat or vegetables because they consider mushrooms as a natural product that does not have chemicals as Alonso-Aguilar et al. [23] reported.

Economic Index (EI). *Amanita basii* has the highest price ($60–$80 Mexican pesos per four basidiomata), but only a few people sell it. A 15 l bucket full of *Fistulinella wolfeana* was priced at $35. Other species, such as *Lactarius indigo*, *Ramaria* spp., *Leccinum* aff. *aurantiacum* (Bull.) Gray, *Hypomyces lactifluorum*, *Boletus variipes*, *B. auripes*, *Agaricus campestris*, *Calvatia cyathiformis*, *Cantharellus cibarius*, and *Armillaria mellea* (Vahl) P. Kumm. have a lower value, between $15 and $50 for about ten basidiomata. *Calvatia cyathiformis* is priced at $70, and *A. campestris* costs $50, but this can only be collected on plains at the beginning of the rainy season. To compare prices, at Acambay market (State of México), fungi prices range from $40 to $80 per 200 to 400 g for species like *B. variipes* and *A. caesarea* (Scop.) Pers. complex, while in the municipal market of Amealco (Querétaro), a plate of mushrooms, which contains about 100 g of *C. cibarius* and *Ramaria* spp. has a price of about $20 or 200 g for $35. Many respondents either have a store, work as laborers, have a piece of land to grow corn, or have relatives who send them money to help cover expenses, thus collecting mushrooms is not their main economic source. Only some people trade mushrooms for basic supplies like corn seeds, oil, beans, rise, etc. Until now, there is no consensus about this sub-index because it is in function of almost all the sub-indexes that compound the EMCSI and could be disregarded [23]. In this case, even with economic potential [22], people prefer to consume the mushrooms over selling them.

The information obtained in the three communities and the value of the indexes and total EMCSI are shown in Table 3. The three main mushrooms were *L. indigo* (63.24), *A. basii* (61.95), and *F. wolfeana* (61.23). It is probably because *L. indigo* and *F. wolfeana* are available after the rainy season, which gives them a high value, even over *A. basii*. Some people consume them for up to 1 year after they have been collected; they put dry basidiomata in hot water and then cook them. This contrasts with QI and PAI values, where *A. basii* had a higher value in the QI and a lower value in the PAI, compared to *L. indigo* and *F. wolfeana*. In many cases, before we start talking about mushrooms, people mention *A. basii* immediately, so we might consider these three species with similar ethnomycological importance values, and this matches with the results of other studies [22, 23], where *A. caesarea* complex are the most priced mushrooms, although it is possible to find other more valuable species in other places [12, 16, 28].

### Uses

Edibility was the main use found for mushrooms. Almost all respondents mentioned that they consume fungi, only three people said they quitted eating them or using them for reasons discussed below. Regarding medicinal use, only three people mentioned *Ustilago maydis* as a remedy for burns and to combat vomiting. Mature basidiocarps are used by placing them directly on burn injuries, where informants argue a faster healing. This coincides with reports from other cultures that use *U. maydis* as a remedy for burns [28]. Other informants report that people use this species as a remedy when horses do not want to drink water, so they give them the mushroom in a solution of water with spores, and the horses drink water again. This might be the first veterinary report for mushrooms, and therefore, further research on this aspect is necessary.

Some informants mentioned the use of *E. frostii* to control diabetes. Basidiomes of *Leccinum* spp., which are not consumed, are used by some people as toys during harvest time, throwing these ones to others as a game. Mushrooms that they do not consume are considered poisonous, even if others eat them. A recent record of mushroom poisoning occurred in a community near Amealco, where four people confused the *Omphalotus mexicanus* Guzmán & V. Mora with a *Lactarius indigo*, and the symptoms consisted mainly of vomit and diarrhea; they were treated at the health center only with activated carbon and antihistamines, and people mentioned that one woman and her daughter died about 10 years ago due to the consumption of poisonous mushrooms; however, they do not know which species. According to Peña-Cañón and Enao-Mejíar [26], people only eat species they are sure to know.

People harvest mushrooms provided by the forest as part of the natural resources available during the rainy season. On the other hand, many of them used to grow vegetables a couple of months before the rainy season began, so they could get benefits from mushroom collection, by adding diversity and enrichment of the daily diet and helping them to save money. Mushroom recollection was restricted mainly to August and September, the months where we conducted the ethnographic work and where we found most of the species. All mushrooms have a utilitarian category [50]. We suspect in this particular study that they do not have a hierarchical category as Berlin [51] proposed for plants and animals because informants usually just named and used mushrooms related to their daily use, such as *Russula* spp. which is called “Hongo de Borrego (a)” (lamb mushroom), because when people take their animals out to the pasture, lambs eat these mushrooms. *C. cibarius* is called “Pericón” or “Santa María” due to the similar yellow color with *Tagetes lucida* Cav. Other example included *L. indigo*, which has names related with its bright blue color “pant mushroom,” because the blue color is a reminder of a pair of blue jeans. As mentioned, people only named mainly edible mushrooms and considered the remaining non-used as venomous. It is interesting how they recognize the good ones, and this knowledge is transmitted generationally mainly by women, being the principal characteristics color, smell, maturity, and in some cases, the taste (they avoid spicy and bitter flavors). Alonso-Aguilar et al. [23] found *A. basii*, among other mushrooms, is the most appreciated species. In the present work, we found *F. wolfeana*, *L. indigo*, *Ramaria* spp., and *B. variipes* (species close to *B. pinophilus* Pilát & Dermek) as having the highest values of EMCSI, and the species had highest sub-indexes values. These values are in function of the QI sub-index because not all the interviewed people mentioned other species with significant abundances, such as *Russula* aff. *brevipes*, *E. frostii*, *Lyophyllum* sp., *G. dryophilus*, *A. novinupta*, or *H. crispa*, which were mentioned by less than 20% of the interviewed people. In this case, those positions in the EMCSI value are relative to the mentioned above and could be modified along the time by the factors mentioned in KTI sub-index discussion.

Finally, we have noted that some people begin to get upset with the intervention of people who do not belong to their community; for this reason, it is important to consider as necessary a previous diagnose, and then get previous consent, considering and giving all the people the opportunity to participate and being interviewed, as observed by Garibay-Orijel et al. [22], and according to Ford [52], it is necessary to recognize them as authors of any work that involves traditional knowledge, not only on mushrooms. On the other hand, most of the people do not have any problem with sharing their knowledge, and in some cases, we developed a friendly relationship that persists even after the work has been finalized.

### Cluster of similarities

**Fig. 2:**
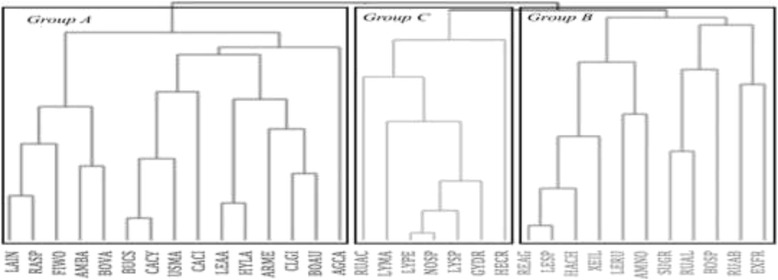
Clustering tree showing the major groups of mushrooms according to their Euclidean distance. LAIN, *Lactarius indigo*; AMBA, *Amanita basii*; FIWO, *Fistulinella wolfeana*; RASP, *Ramaria* sp.; BOVA, *Boletus variipes*; BUCS, *Boletus* aff. *speciosus*; LEAA, *Leccinum* aff. *aurantiacum*; HYLA, *Hypomyces lactifluorum*; CACY, *Calvatia cyathiformis*; ARME, *Armillaria mellea*; USMA, *Ustilago maydis*; CLGI, *Clitocybe gibba*; AGCA, *Agaricus campestris*; RUAC, *Russula* aff. *cyanoxantha*; REAG. *Retiboletus* aff. *griseus*; BOUA, *Boletus auripes*; LESP, *Leccinum* sp.; SUGR, *Suillus granulatus*; RUAB, *Russila* aff. *brevipes*; BOSP, *Boletus* sp.; HARCH, *Harrya chromapes*; LYMA, *Lycoperdon marginatum*; LYPE, *Lycoperdon perlatum*; EXFR, *Exudoporus frostii*; CACI, *Cantharellus cibarius*; NOSP, *Nolanea* sp.; RUAE, *Russula* aff. *emetica*; LYSP. *Lyophyllum* sp.; LERU. *Leccinum rugosiceps*; XEIL. *Xerocomus illudens*; GYDR. *Gymnopus dryophilus*; AMNO. *Amanita novinupta*; HECR. *Helvella crispa*

The dendrogram of similarities (Fig. 2) with the standardized Euclidean distances (SED) linkage of 7.15 shows that the biggest group (A) is composed by *L. indigo*, *Ramaria* spp., *F. wolfeana*, *A. basii*, *B. variipes*, *B*. cf. *speciosus*, *C. cyathiformis*, *U. maydis*, *C. cibarius*, *L.* aff. *aurantiacum*, *H. lactifluorum*, *A. mellea*, and *Clitocybe gibba* (Pers.) P. Kumm., and includes *B. auripes* and *C. cibarius*. *U. maydis* has no EI value but has a high TSAI value because it is one of the most palatable mushrooms. In this group, the closer species were *B.* cf. *speciosus* and *C. cyathiformis* (with a linkage SED = 0.65). The second group (B) is formed by *Retiboletus* aff. *griseus* (Frost) Manfr., *Leccinum* sp., *H. chromapes*, *X. illudens*, *Leccinum rugosiceps* (Peck) Singer, *A. novinupta*, *S. granulatus*, *R.* aff. *lepida*, *Boletus* sp., *R.* aff. *brevipes*, and *E. frostii*, species that show medium to low QI values; and the third group (C) is composed of *R*. aff. *cyanoxantha*, *L. marginatum*, *L. perlatum*, *Nolanea* (Fr.) P. Kumm., *Lyophyllum* sp., *G. dryophilus*, and *H. crispa*, the last is the one with the lowest EMCSI value. This group had a linkage SED of 3.96.

### Multidimensional scaling and principal components analysis

**Fig. 3:**
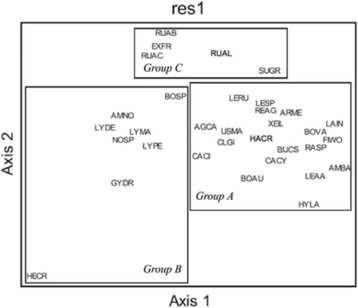
Similarity of species grouped as a result of the MDS analysis. LAIN, *Lactarius indigo*; AMBA, *Amanita basii*; FIWO, *Fistulinella wolfeana*; RASP, *Ramaria* sp.; BOVA, *Boletus variipes*; BUCS, *Boletus* aff. *speciosus*; LEAA, *Leccinum* aff. *aurantiacum*; HYLA, *Hypomyces lactifluorum*; CACY, *Calvatia cyathiformis*; ARME, *Armillaria mellea*; USMA, *Ustilago maydis*; CLGI, *Clitocybe gibba*; AGCA, *Agaricus campestris*; RUAC, *Russula* aff. *cyanoxantha*; REAG, *Retiboletus* aff. *griseus*; BOUA, *Boletus auripes*; LESP, *Leccinum* sp.; SUGR, *Suillus granulatus*; RUAB, *Russila* aff. *brevipes*; BOSP, *Boletus* sp.; HARCH, *Harrya chromapes*; LYMA, *Lycoperdon marginatum*; LYPE, *Lycoperdon perlatum*; EXFR, *Exudoporus frostii*; CACI, *Cantharellus cibarius*; NOSP, *Nolanea* sp.; RUAE, *Russula* aff. *emetica*; LYSP, *Lyophyllum* sp.; LERU, *Leccinum rugosiceps*; XEIL, *Xerocomus illudens*; GYDR, *Gymnopus dryophilus*; AMNO, *Amanita novinupta*; HECR, *Helvella crispa*

**Fig. 4:**
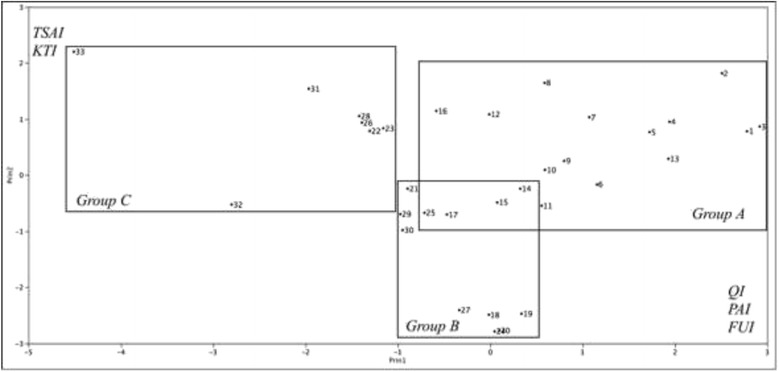
Principal component analysis obtained from similarities in EMCSI values of mushrooms used in Tesquedó, Xajay, and Tenasdá communities, Amealco, Querétaro, México. Table 3 indicates the numbered species

An analysis of the stress in the MDS suggested a 3D configuration (mean = 3.859 and *P* = 0.0196). According to the cluster shown in Fig. 2, mushrooms appear in three main groups A, B, and C (Fig. 3). In this case, we can see a slight difference in the species that conform all groups. The six main mushrooms (and at least the majority of the rest of the species that conform this group) are contained in group A, while the species from groups B and C appear mismatched. These results are supported by the PCA (Fig. 4). The first three principal components explain the 70.93% of the variance. PC1 variables were FUI (0.538), PAI (0.476), and QI (0.474; PC2 variables were TSAI (0.551) and KTI (0.489); and PC3 variables were HI (0.497) and MFFI (− 0.477). We only consider PC1 and PC2 because they are strongly related; frequency of use of sp_i_ is directly related to the abundance of sp_i_, and this is influenced by the number of mentions of sp_i_, as we can see in PCA. The last components, which conform PC3, have both positive and negative values, which makes sense due to only a few species that cause stomach ache, such as *Suillus* spp. or red spicy cap *Russula* spp., but these species are considerably abundant.

These results make sense if we consider the history behind those species; in all cases, people know them due to their families, which mean that these species have been used for almost three consecutive generations. The relation in PC2 variables is in function of the tradition of consuming the preferred species over the fewer accustomed ones by the main transmitters of knowledge; new collectors learn how to recognize all of them, but making emphasis on the preferred species. This contrasts with the PC1 components since the preference over a specific mushroom depends on its frequency of use and the abundance.

**Fig. 5:**
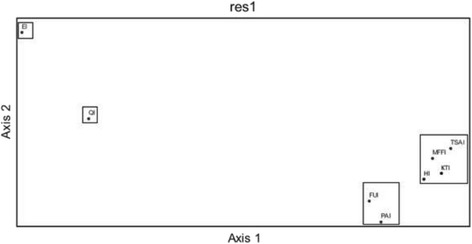
Principal components after comparing the results of the sub-indexes. EI, Economic Index; FUI, Frequency of Use Index; QI, Mention Index; KTI knowledge transmission index; HI, Health Index; TSAI, taste score appreciation index; PAI, perceived abundance index; MFFI, multifunctional food index

Figure 4 shows the similarities between species related, as a result of the values of every sub-index. This has a similar tendency to MDS analysis and PCA for sub-indexes (Fig. 5), wherein both cases, main species are affected by variables of the PC1 and PC2.

Alonso et al. [14] reported a similar tendency in their results, observing the same six main species we found in a similar environment, and as observed by Garibay-Orijel et al. [22], these mushrooms could be found on forest mainly composed of *Quercus* too, but with different values as we see in this survey. Although, it might be a tendency in temperate zones with their main species [16, 22, 23]. In this case, human communities are very close to the forest, so people can easily collect mushrooms and use them as a food source, which is the main use category. *Agaricus campestris*, *C. cyathiformis*, and *U. maydis* are out of the forest but have an important role, where the first two species are the first available in the season, and the last one depends on corn planting. *Lactarius indigo*, *A. basii*, and *F. wolfeana* are the main species found in this survey, among 33 different mushrooms. Although kids participate in mushroom harvesting, many of them do not like mushrooms. Bautista [29] refers the preference of new tendencies, so that means erosion in the ethnomycological knowledge; the situation also observed in the *Hñähñu* (Otomí) language. On the other hand, people refer that forest does not have the same shape they remember, and many of them mentioned that the amount of mushrooms has decreased because of environmental degradation and deforestation.

## Conclusion

The current state of knowledge in the studied communities is strong but with a tendency to disappear due to migration and less interest among new generations. Mushroom consumption is part of integral and seasonal cultural knowledge of the resources in the Otomí communities at Amealco municipality in Querétaro, México. Mainly women transmit traditional mushroom knowledge. Food, medicine, and venom were the three principal categories we identified, and only few people use them for trade or sale within the same communities. The use of *U. maydis* as a veterinary medicine could open the possibility for research into animal welfare products. The uses of quantitative methods such as the Edible Mushrooms Cultural Significant Index (EMCSI) prove an easy and important method for future studies. The socio-politic conditions have been changing, and the economy has shown modifications over the last years. Therefore, we consider that future studies must be conducted using compatible methods in order to compare future results and then analyze the tendencies of traditional knowledge over time.

## Appendix

**Table 4:** List of the species and its corresponding voucher

Species	Voucher
*Agaricus campestris*	Robles 331
*Amanita basii*	Robles 8
*Amanita novinupta*	Robles 25
*Armillaria mellea*	Robles 50, 289
*Boletus auripes*	Robles 268
*Boletus* cf. *speciosus*	Robles 222
*Boletus variipes*	Robles 13, 19, 261, 267
*Boletus* sp.	Robles 7
*Calvatia cyathiformis*	Robles 332
*Cantharellus cibarius* complex	Robles 137
*Clitocybe gibba*	Robles 29, 148
*Exudoporus frosti*	Robles 32, 260
*Fistulinella wolfeana*	Robles 10, 162
*Gymnopus driophilus*	Robles 100A, 263
*Harrya chromapes*	Robles 128, 285
*Helvella crispa*	Robles 99
*Hypomyces lactifluorum*	Robles 468
*Lactarius indigo*	Robles 515
*Leccinum* aff. *aurantiacum*	Landeros 3465
*Leccinum rugosiceps*	Robles 122, 287
*Leccinum* sp.	Robles 35
*Lycoperdon marginatum*	Robles 26
*Lycoperdon perlatum*	Robles 281
*Nolanea* sp.	Robles 3
*Ramaria* spp.	Robles 9
*Retiboletus* aff. *griseus*	Robles 45
*Russula* aff. *brevipes*	Robles 24
*Russula* aff. *cyanoxantha*	Robles 129
*Russula* aff. *lepida*	Robles 11
*Suillus granulatus*	Robles 12
*Ustilago maydis*	Robles 667
*Xerocomus illudens*	Robles 127

## Abbreviations

EI: Economic Index
EMCI: Edible Mushrooms Cultural Significant Index
FUI: Frequency of Use Index
HI: Health Index
KTI: Knowledge transmission index
MDS: Multidimensional scaling
MFFI: Multifunctional food index
PAI: Perceived abundance index
PC1, 2, 3: Principal component one, two, three
PCA: Principal components analysis
QI: Quotation Index
SED: Standardized Euclidean distances
TSAI: Taste score appreciation index
